# Plasmodium palmitoylation machinery engineered in *E. coli* for high‐throughput screening of palmitoyl acyl‐transferase inhibitors

**DOI:** 10.1002/2211-5463.12564

**Published:** 2019-01-10

**Authors:** Preeti Yadav, R Ayana, Swati Garg, Ravi Jain, Raj Sah, Nishant Joshi, Soumya Pati, Shailja Singh

**Affiliations:** ^1^ Special Centre for Molecular Medicine Jawaharlal Nehru University New Delhi India; ^2^ Department of Life Sciences School of Natural Sciences Shiv Nadar University Greater Noida, Uttar Pradesh India

**Keywords:** acyl‐biotin exchange, click chemistry, drug screening, *E. coli*, palmitoylation, PfDHHCs

## Abstract

Lipid‐based palmitoylation is a post‐translation modification (PTM) which acts as a biological rheostat in life cycle progression of a deadly human malaria parasite, *Plasmodium falciparum*. *P. falciparum* palmitoylation is catalyzed by 12 putative palmitoyl acyl‐transferase enzymes containing the conserved DHHC‐CRD (DHHC motif within a cysteine‐rich domain) which can serve as a druggable target. However, the paucity of high‐throughput assays has impeded the design of drugs targeting palmitoylation. We have developed a novel strategy which involves engineering of *Escherichia coli*, a PTM‐null system, to enforce ectopic expression of palmitoyl acyl‐transferase in order to study *Plasmodium*‐specific palmitoylation and screening of inhibitors. In this study, we have developed three synthetic *E. coli* strains expressing *Plasmodium*‐specific DHHC proteins (PfDHHC7/8/9). These cells were used for validating acyl‐transferase activity via acyl‐biotin exchange (ABE) and clickable chemistry methods. *E. coli* proteome was found to be palmitoylated in PfDHHC‐expressing clones, suggesting that plasmodium DHHC can catalyze palmitoylation of *E. coli* proteins. Upon treatment with generic inhibitor 2‐bromopalmitate (2‐BMP), a predominant reduction in palmitic acid incorporation is detected. Overall, these findings suggest that synthetic *E. coli* strains expressing PfDHHCs can enforce global palmitoylation in the *E. coli* proteome. Interestingly, this finding was corroborated by our *in silico* palmitoylome profiling, which revealed that out of the total *E. coli* proteome, 108 proteins were predicted to be palmitoylated as represented by the presence of three cysteine consensus motifs (cluster type I, II, III). In summary, our study reports a proof of concept for screening of chemotherapeutics targeting the palmitoylation machinery using a high‐throughput screening platform.

**Abbreviations**
2‐BMP: 2‐Bromo palmitate
ABE: acyl‐biotin exchange
DHHC domain: Asp‐His‐His‐Cys domain
GST: Glutathione‐S‐transferase
IPTG: Isopropyl β‐D‐1‐thiogalactopyranoside
MSA: multiple sequence alignment
ODYA: Octadecynoic Acid
OGA: Oregon green azide
PATs: palmitoyl acyl‐transferases
PfDHHC: *Plasmodium falciparum* DHHC or palmitoyl acyl‐transferase enzyme
PTMs: post‐translational modifications
TCEP: Tris(2‐carboxy‐ethyl) phosphine

Post‐translational modifications (PTMs) play a crucial role in the biology of any cell by increasing the functional diversity of the proteome 1, 2. Such modifications can change the property of any protein according to the developmental stages and physiological conditions. These include covalent addition or removal of low‐molecular‐weight groups such as acetylation, amidation, biotinylation, glycosylation, myristoylation, palmitoylation, and phosphorylation. Among these, palmitoylation involves thioesterification of C‐16 fatty acyl moieties at specific cysteines and is known to act as a biological rheostat for cellular homeostasis, subcellular trafficking and protein–protein interactions 3. This process is reversible and catalyzed by palmitoyl acyl‐transferases (PATs or termed as DHHCs), which contains the canonical Asp‐His‐His‐Cys (DHHC) domain.

Recent advances suggest PATs can be putative drug targets for parasitic diseases such *Leishmaniasis* and *Toxoplasmosis* 4, 5, 6. However, among these protozoan parasitic diseases, malaria still remains to be the leading cause of death in tropical areas and increasing global incidence. Even though several established drugs are in clinical use for malaria treatment, there is an obstacle in therapy due to drug resistance.

*Plasmodium falciparum*, an intracellular parasite belonging to phylum Apicomplexa, is responsible for the most severe form of malaria. Its ultrastructure comprises of a pellicle containing the plasma membrane and inner membrane complex (IMC). IMC is responsible for the gliding motility or locomotion of the parasite necessary for host cell invasion. Many proteins of IMC undergo several PTMs such as palmitoylation to regulate crucial biological processes including motility, localization, and stage‐specific activity. In case of *Plasmodium falciparum*, the causative agent of malaria approximately 10% of the proteins has been identified to be palmitoylated in the schizont (infective) stage exclusively 7. This percentage might vary based on the data collation from other developmental stages of the parasite. Recent studies have shown that palmitoylation and DHHC enzymes can play crucial roles in both the intra‐erythrocytic and gametocytic stages 5, 8. The parasite encodes for 12 DHHC (PfDHHC) proteins that are localized to different membrane compartments and facilitates transfer of palmitate group to target proteins. Based on the proteomics data from PlasmoDB and existing literature, it is evident that PfDHHC7 and PfDHHC9 have been characterized in the schizont stages 5, 8. While PfDHHC9 has been detected in the schizont stage and has implications in gametocytogenesis, PfDHHC7 has been found to be localized to the rhoptry organelles. However, schizont stage protein PfDHHC8 is yet to be fully characterized. Based on this, we have characterized PfDHHC7/8/9 and specifically emphasized on PfDHHC8 expression as a proof of concept for designing an assay system capable of screening novel small molecules against malaria.

Previous methods to quantify and detect palmitoylation are not foolproof. The most frequently used protein palmitoylation assays involve the metabolic labeling of cultured cells with radioactive forms of palmitate, such as [3‐H] palmitate which could detect dynamic palmitoylation/depalmitoylation of full‐length proteins based on pulse‐chase analyses 9. Due to handling of radioactivity involved in multiple steps, this method became obsolete. Another method known as acyl‐biotin exchange (ABE) is used to purify the palmitoylome and involves removal of lipid moiety from proteins following treatment with hydroxylamine. This shifts the mass of the depalmitoylated peptide thus, making it easy to detect palmitoylation 10. However, due to the tedious nature of the protocol, it is unfavorable for screening large compound libraries. An alternate method to study protein palmitoylation is fatty acyl exchange chemistry, wherein a fatty acid group on the palmitoylation site is exchanged for a more readily detectable label such as thiol‐specific reagents 11, 12. Due to its low‐throughput nature, it is not favorable for drug discovery assays. Another technique termed as *in vitro* palmitoylation employs fluorescently labeled lipopeptides to the samples containing the enzyme source, and the reaction is initiated by the addition of unlabeled palmitoyl‐CoA. Although this assay is much faster than other palmitoylation assays, the use of high‐performance liquid chromatography (HPLC) for detection makes it less throughput for drug screening.

Toward this, we thought to innovate a simple, affordable, and high‐throughput DHHC enzyme‐specific platform which can revolutionize screening antiparasitic drugs. Since the prokaryotic cells inherently lack the palmitoylation machinery, we designed synthetic *Escherichia coli* expressing PfDHHC7/8/9 to study their activity *ex vivo*, mimicking the *Plasmodium*‐specific palmitoylation machinery. Interestingly, the *E. coli* proteome demonstrated differential palmitoylation, as evident by both click chemistry and ABE‐based studies. We could further demonstrate that acyl‐transferase activity of PfDHHC8 is very specific and sensitive to 2‐Bromo palmitate (2‐BMP), a known inhibitor of palmitoylation. Further *in silico* analysis of *E. coli* proteome revealed cysteine specific palmitoylation sites in 108 proteins predicted to be palmitoylated. Overall, this study represents a novel high‐throughput platform for screening DHHCs of any organism (here we have shown *P. falciparum*) or novel palmitoylation inhibitors using a relatively PTM‐null engineered bacterial system.

## Methods

### Sequence analysis of PfDHHCs

Using various databases like the PlasmoDB, PiroplasmaDB, and UniProt, we extracted DHHC sequences of different organisms including *Plasmodium falciparum*,* Plasmodium berghei Toxoplasma gondii*,* Babesia bovis*,* Theileria parva*,* Cryptosporidium muris,* and *Eimeria tenella*. This data exploration was done using different apicomplexan‐specific databases including plasmodium genomics resource database (PlasmoDB) 13, toxoplasma genomics resource database (ToxoDB) 14, piroplasma genomics resource database (PiroplasmaDB) 15, and UniProt 16 for fetching other organism‐specific protein sequences. Using PlasmoDB, putative DHHC gene sequences in *P. falciparum* (PfDHHC) were annotated based on conserved domain architecture. The domain architecture of protein sequences was assessed via SMART‐Batch and INTERPRO online servers 17, 18. We performed multiple sequence alignment (MSA) of the 12 annotated PfDHHCs using MUSCLE algorithm (implemented in jalview software, Elixir‐UK, https://elixir-europe.org) 19. These 12 PfDHHCs along with PAT sequences from six other organisms namely closer members *P. berghei* and *T. gondii*, and distant members of Apicomplexa namely *B. bovis*,* T. parva*,* C. muris,* and *E. tenella* were further used to construct a neighbor‐joining (NJ) tree. The bootstrap consensus tree inferred from 500 replicates finally represented the evolutionary history of the taxa analyzed using mega7 software (Pennsylvania State University, University Park, PA, USA) 20. The evolutionary distances were computed using the Dayhoff matrix based method and are in the units of the number of amino acid substitutions per site.

### Docking studies of PfDHHCs

The sequences of 12 PfDHHCs were obtained from PlasmoDB and their structural models were constructed using the I‐TASSER web server using template as HsDHHC20 (PDB code: 2BML) 21. Using modrefiner software (University of Michigan, Ann Arbor, MI, USA), the structures were further refined for docking with substrate palmitic acid (PA) and a known inhibitor of palmitoylation, 2‐BMP 22. Quality validation of the resultant models was done with RAMPAGE. The theoretical models were visualized with pymol Molecular Graphics System (Schrödinger, LLC, New York, NY, USA), version 1.7.4 23. Chemical structure of 2‐BMP was downloaded from PubChem database in SDF format, converted to standard PDB format and energy minimized using Chem3D Pro 12.0. Molecular docking was performed by using AutoDock Vina 24 to rationalize the activity of PA and 2‐BMP against all 12 PfDHHCs. As per the already established 2‐BMP binding pocket in PfDHHC homologue, HsDHHC20 (PDB ID: 6BML), we ensured that the active site residues were covered while constructing the virtual grid for docking. Incorporating the predicted ligand binding groove, a virtual 3D grid of mean (20 Å) × mean (20 Å) × mean (20 Å) with varying *x*,* y*,* z* coordinates of the center of energy was constructed for individual PfDHHCs through the Autogrid module of AutoDock Tools 24. We performed molecular docking studies using AutoDock Vina with compounds to rationalize its activity 25. The top‐ranked conformations of compound within the protein catalytic pocket were selected based on the lowest free binding energies. The stable conformations were visualized for polar contacts like hydrogen bonds. Top scoring docked conformations of the scaffold were selected based on the most negative free binding energies and visualized for polar contacts with the amino acid residues of PfDHHC1‐12 by using pymol Molecular Graphics System 23.

### Culture of *E. coli* strain

*Escherichia coli* strain C43 was used, and the primary culture was done at 37 °C, 16 h in LB (Himedia, Mumbai, India) media. Secondary culture was done in Terrific Broth (Sigma, Ronkonkoma, NY, USA) containing 50 μm ZnCl_2_ (Sigma). Induction was given by 1 mm Isopropyl β‐D‐1‐thiogalactopyranoside (IPTG) (SRL) at 16 °C for 22 h. Octadecynoic acid (ODYA)‐palmitic acid (Cayman Chemicals, Ann Arbor, MI, USA) was added during induction in both uninduced and induced samples. In the inhibitor‐treated sample, 2‐BMP (Sigma) was added at the time of induction.

### Click chemistry

Palmitoylation was examined by labeling with Click Tag™ Palmitic Acid Alkyne, a 16‐carbon saturated fatty acid group combined with a specific azide dye‐Oregon Green® 488 (Invitrogen, Carlsbad, CA, USA) dye as described in our recently published article 6. Briefly, palmitic acid (Alk‐C16, Cayman Chemicals) was dissolved in DMSO to achieve final stock concentration of 50 mm. Bacteria were washed twice with cold PBS, fixed with chilled methanol for 5 min, and further permeabilized using 0.01% Triton X‐100 (Sigma) in water at RT for 5 min. These processed bacterial samples were subjected to a click labeling reaction in 100 μL of dye mix containing 0.1 mm azide dye (Oregon Green 488‐azide, Thermo Scientific, Waltham, MA, USA), 1 mm Tris‐(2‐carboxyethyl)‐phosphine hydrochloride (TCEP‐Sigma), and 1 mm CuSO_4_ (Sigma) in PBS for 1 h. After incubation, bacteria were pelleted down and washed twice by 1X PBS. For microscopy, a smear was made which was mounted in antifade mounting solution. Images were acquired by fluorescence microscope (Zeiss, Oberkochen, Germany). Samples were also examined by flow cytometry (FACS‐BD LSR II Fortessa) by resuspending the final pellet in 1X PBS (200 μL). Analysis was done by flowjo software (FlowJo LLC, Ashland, OR, USA). Fluorometric estimation (100 μL sample in 1X PBS) using fluorimeter (Varioskan, Biotek Instruments, Winooski, VT, USA) was also done after normalization of O.D. at 600 nm.

### *E. coli* palmitoylome purification using acyl‐biotin exchange method

Acyl‐biotin exchange purification of whole bacterial lysate was carried out using the established method as published by Wan and colleagues, with the following modifications 26. Bacteria were resuspended in 400 μL of lysis buffer containing 10 mm NEM. The rest of the procedure was performed as described 6, 26. Samples were run on 12% SDS gel, and bands were visualized by silver staining.

### PfDHHC7/8/9 specific cloning and expression

PfDHHC7 gene's 360‐bp domain was PCR‐amplified using primers: Fwd 5′‐CGCGGATCCTTGAAGAGAGGAATTATTGAG ‐3′ and Rev 5′GTGCGTCGACTATTGAACAGTATATTAAAGATAACA ‐3′. Whole length of 942‐bp PfDHHC8 gene was truncated from N and C terminal by 26 and 25 nucleotides, respectively, resulting in a 789‐bp region. This was PCR‐amplified using primers: Fwd 5′‐CGCGGATCCTGTAAAGGAAATATCATAACAGGGCCT‐3′ and Rev 5′ATGCGTCGACTTACCATTCGACTTGGATTTTATCCACGG‐3′. 882‐bp‐long PfDHHC9 was PCR‐amplified using following primers: Fwd 5′‐CGCGGATCCATGAATAATTATTTGGCATTTATC‐3′ and Rev 5′‐5′ATGCGTCGACTTAATCTCCATCTTCCTTTATG‐3′. These genes were cloned into pGEX‐4T‐1 vector (GE Healthcare, Chicago, IL, USA) at Bam‐HI and Sal‐I sites and expressed in *E. coli* C43 cells. The resultant clones were labeled as PfDHHC8 and PfDHHC9, respectively. Transformed C43 cells were induced for expression of recombinant proteins tagged with Glutathione‐S‐transferase (GST) using 1 mm IPTG at 16 °C for 22 h. Supernatant and pellet were separated. Protein was obtained in the supernatant fraction.

### Immunoblotting

Supernatant was loaded onto polyacrylamide gel (12%), and western blotting was done using an anti‐GST antibody according to a standard protocol.

### *In vitro* culture of *P. falciparum*

Frozen stocks of *P. falciparum* 3D7 strain were thawed and cultured using a modified method from Garg *et al*. 27, 28. Parasites were maintained in complete RPMI (RPMI 1640, Invitrogen), supplemented with 50 mg·L^−1^ hypoxanthine (Sigma), 5 gm·L^−1^ Albumax I (Gibco, Invitrogen, Waltham, MA, USA), and 2 gm·L^−1^ sodium bicarbonate (Sigma) and O+ human erythrocytes, under mixed gas conditions (5% O_2_, 5% CO_2,_ and 90% N_2_) at 37 °C.

### Growth inhibition assay in *P. falciparum* using 2‐bromopalmitate

To assess the effect of 2‐BMP on growth rate of parasite, synchronized parasite culture at trophozoite stage was treated with increasing concentration of 2‐BMP (25–200 μm) and allowed to grow over one cycle, that is, 48 h. After one cycle, the parasite was freeze‐thawed and processed for SYBR green staining. Briefly, equal volume of lysis buffer containing Tris (20 mm; pH 7.5), EDTA (5 mm), saponin (0.008%; W/V), and Triton X‐100 (0.08%; V/V) is added to parasite culture. 1X SYBR green dye (Invitrogen) was added to the parasite and incubated for 3 h at 37 °C. The plate was read by fluorimetry at excitation and emission wavelength of 485 and 530 nm, respectively. Percent growth inhibition was calculated using the following formula:

%Growth Inhibition=(Control‐treated)/Control∗100

### Colony‐forming unit assay

*Escherichia coli* C43 strain containing the PfDHHC8 was cultured according to the conditions mentioned previously. Induction was done using 1 mm IPTG. After every 2 h, serial dilutions were made from uninduced, induced, and inhibitor‐treated sets. Only three dilutions were used for plating on the LB plates containing Ampicillin. For plating, 5 μL of culture was used.

### Prediction of global palmitoylome of *Escherichia coli*

We used the *E. coli* proteomics dataset deposited under Proteomics module of UniProt database 16. The total proteome was scanned for potential palmitoylation sites using a group‐based prediction system software (gps‐lipid 1.0, OmicX, Seine Innopolis, Le‐Petit‐Quevilly, France) 29. This updated stand‐alone software is based on integration of the particle swarm optimization with an aging leader and challengers (ALC‐PSO) algorithm 29 which is optimized specifically for the prediction of four classes of lipid modifications namely, S‐palmitoylation, N‐myristoylation, farnesylation, and geranylgeranylation. Using a medium threshold cutoff for the palmitoylation predictor module of the software, we could correctly predict five sites as a positive score in individual proteins. To characterize the resultant palmitoylome, firstly we assessed the type of palmitoylation site in individual proteins and dissected them into three types of site clusters as described in the earlier published work from our laboratory 6. Furthermore, we analyzed the frequency of proteins having higher level of palmitoylation to predict the global palmitoylome of *E. coli*.

### Gene ontology (GO) and cellular localization analysis

Gene ontology analysis of all 108 palmitoylated proteins was performed using the UniProt GO server 16. We looked for enriched biological processes and predicted the molecular function and cellular component. We further looked at the distribution of palmitoylated proteins in individual compartments of the *E. coli* to ascertain their functional significance.

### Identification of potential molecular targets and Protein–protein network analysis

Using STRING database, we searched for interacting protein partners to the highly palmitoylated proteins (> 1 palmitoylation site = 108) 30. A stringent cutoff of 0.7 for interaction score was considered for building the primary protein–protein interaction (PPI) network without clustering and no more than 10 primary interactor proteins. This combined confidence score was calculated on the basis of various parameters including phylogenetic co‐occurrence, gene fusion, homology, co‐expression, experimentally determined interaction, and neighborhood on any chromosome which is displayed by the line thickness of edge. Overall, this yielded a strongly enriched PPI network associated with various critical pathways in *E. coli*.

## Results

### PfDHHC proteins exhibit organism‐level conservation within the apicomplexan family

**Figure 1:**
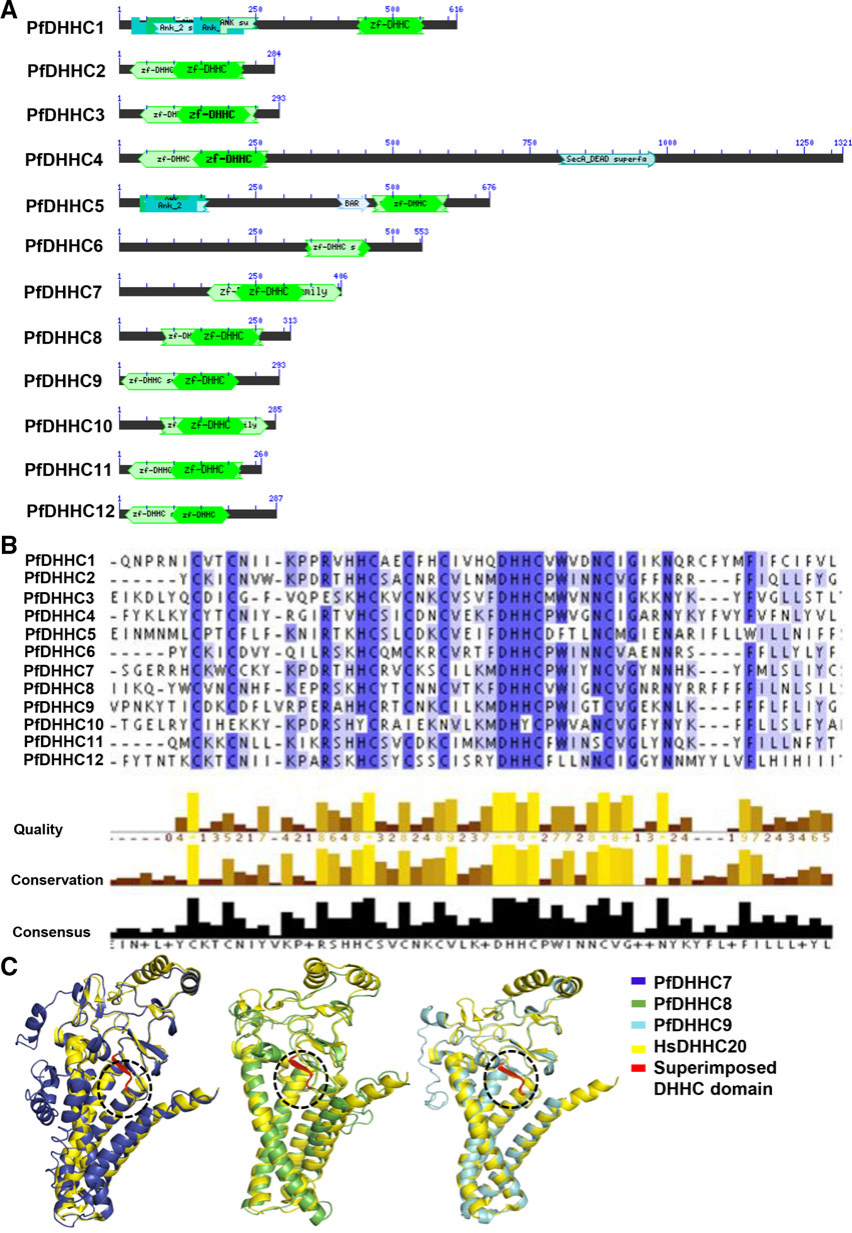
(A) Domain architecture of 12 DHHC domain‐containing gene sequences. (B) MSA showing conservation between *L. donovani* and *T. brucei* palmitoyl acyl‐transferase sequences. (C) Structural alignment of candidate *Plasmodium* DHHCs, namely PfDHHC7, PfDHHC8, and PfDHHC9 with template‐like structure of human DHHC20.

To identify DHHC containing *Plasmodium*‐specific proteins, domain architecture analysis was performed for 12 identified PfDHHC sequences and variable lengths of Asp‐His‐His‐Cys domains (DHHC domains) were observed (Fig. 1A). While numerous transmembrane regions were detected (2–6) without any signal peptide sequences, two of the members, PfDHHC1 and PfDHHC5 proteins, were found to contain Ankyrin repeats (Fig. 1A). MSA analysis showed that the signature DHHC motif is highly conserved in all 12 PfDHHC sequences (Fig. 1B). In addition to the conservation scoring, we also evaluated the occupancy factor (values range from 0 to 1.0) of individual amino acids and found the D‐H‐H‐C sequence was positioned correctly (value = 1.0; Fig. 1B). Additionally, we superimposed the 3D structures of PfDHHC7, PfDHHC8, and PfDHHC9 with homologous human DHHC20 (2BML) to examine the structural similarity between individual DHHC domains (Fig. 1C).

To understand the phylogenetic relationships within this group of proteins, we evaluated their evolutionary proximity to other apicomplexan parasites including closer members *P. berghei* and *T. gondii*, and distant members of Apicomplexa namely *B. bovis*,* T. parva*,* C. muris,* and *E. tenella* (Fig. S1A). The phylogenetic tree was constructed by a bootstrap NJ algorithm. The D‐H‐H‐C motif was found to be highly conserved among all the 61 sequences of DHHC proteins, whereas the whole protein sequences including PfDHHCs showed moderate conservation (Fig. S1A).

### Molecular docking revealed probable competitive binding of palmitic acid and 2‐BMP to catalytic core of PfDHHCs

**Figure 2:**
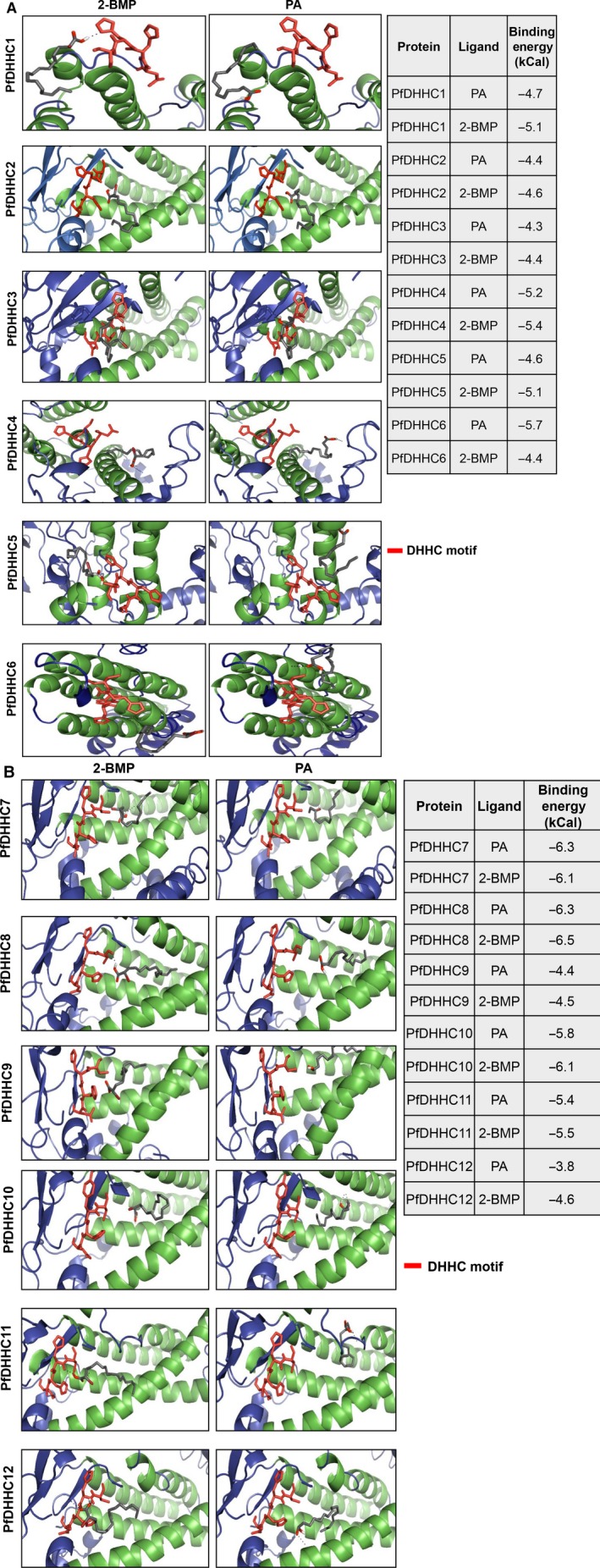
Docking studies of PfDHHC1–12 with the ligand palmitic acid and classical inhibitor 2‐BMP. The representative table shows the binding energy values between the compound and individual DHHC protein.

In order to assess the preferential binding affinity of 2‐BMP/palmitic acid (PA) to each of the 12 *Plasmodium*‐specific DHHC proteins, the drug–protein atomic interactions were examined. Firstly, we constructed structural models of 12 PfDHHCs and these structures were validated using RAMPAGE, with most of the residues were found to be restricted to the favored region (90%) in the Ramachandran plot. Notably, the binding affinity of 2‐BMP to the catalytic pocket was found to be varying from −4.4 to −6.5 kCal·mol^−1^. The bound protein–ligand complex revealed several strong hydrogen bonds specifically between H/H residues within the catalytic pocket and 2‐BMP/PA (2.7–3.4 Å bond lengths), respectively (Fig. 2). In all the protein–ligand models, 2‐BMP showed stronger binding affinity than PA toward PfDHHCs. Notably, an anomaly was observed in case of PfDHHC6 wherein the inhibitor 2‐BMP was observed to bind with lower affinity (−4.4 kCal·mol^−1^) than PA (−5.7 kCal·mol^−1^). Further analysis of bound structures of PfDHHC6 to PA, we found the H‐bond interaction could be detected proximal to the DHHC motif at ASN320 O1/O2 atoms, whereas 2‐BMP was found to be binding farther from the pocket (nonspecifically) to the ASP194 O1/O2 atoms via H‐bonds. We also analyzed the interaction between PfDHHC8 and the enzyme acyl‐intermediate palmitoyl‐CoA and found it to be docking to periphery of the catalytic pocket of the protein (Fig. S1B). The binding energy was recorded to be −6.3 kCal·mol^−1^, and the bulky CoA group was probably hindering the molecule from binding effectively to the DHHC motif area of the pocket.

### *E. coli* expressing PfDHHC proteins did not show significant morphological changes

**Figure 3:**
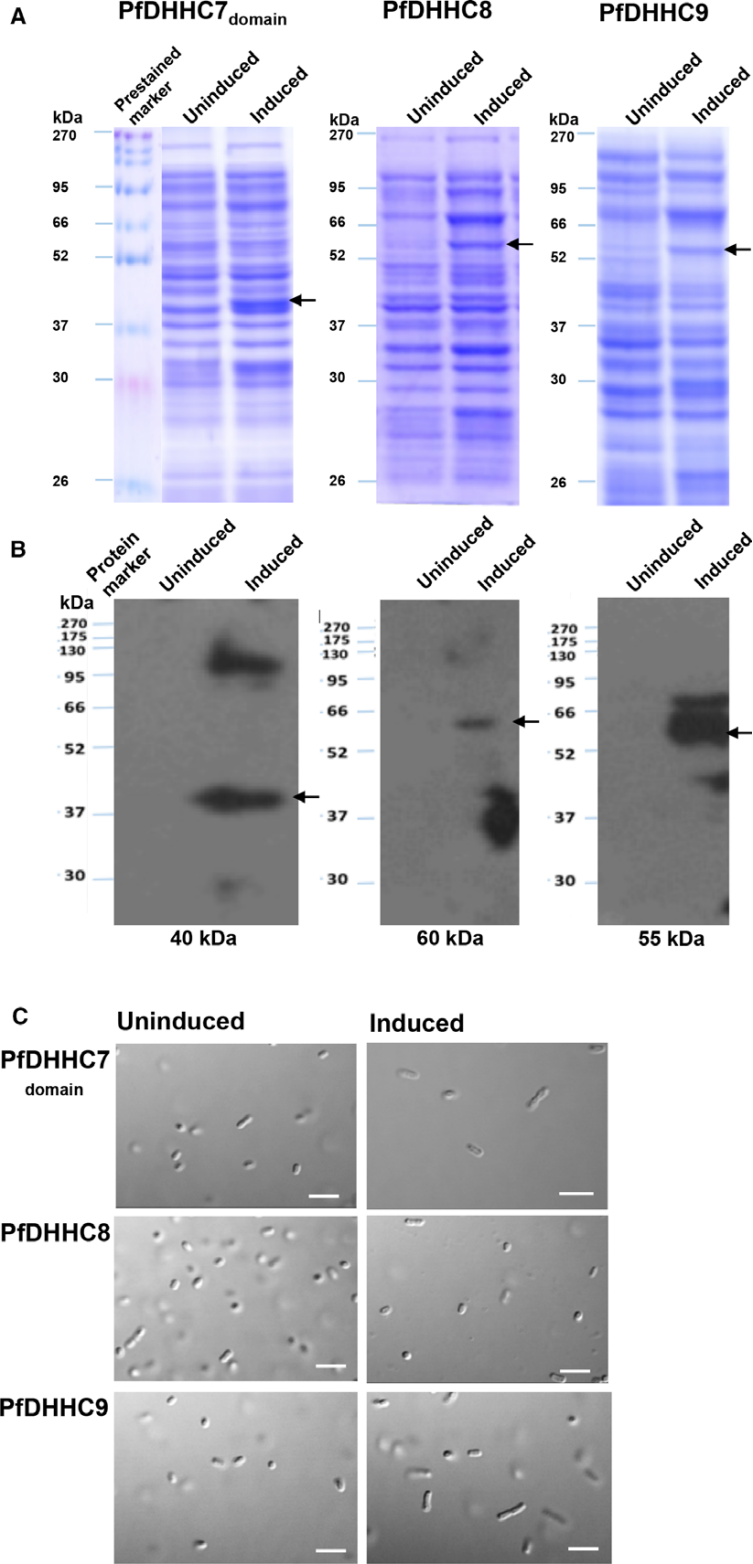
(A) SDS/PAGE analysis of two characterized PfDHHCs in recombinant *E. coli*. (B) Western blot analysis of recombinantly expressed PfDHHCs. (C) Negligible morphological changes were observed in all three engineered *E. coli* cells. Scale bar indicates 1 μm.

The *Plasmodium*‐specific DHHC proteins (PfDHHC7, PfDHHC8, and PfDHHC9) were cloned and expressed in *E. coli*. Using SDS/PAGE, we could detect the protein bands at 40, 60, and 55 kDa for PfDHHC7, PfDHHC8, and PfDHHC9, respectively, and verify their expression by western blot analysis (Fig. 3A,B). Upon induction, no significant change in morphology was observed in case of either of three *E. coli* systems expressing PfDHHC7/8/9 (Fig. 3C).

### Probe‐based clickable chemistry demonstrated palmitoylation in *E. coli* expressing PfDHHC proteins

**Figure 4:**
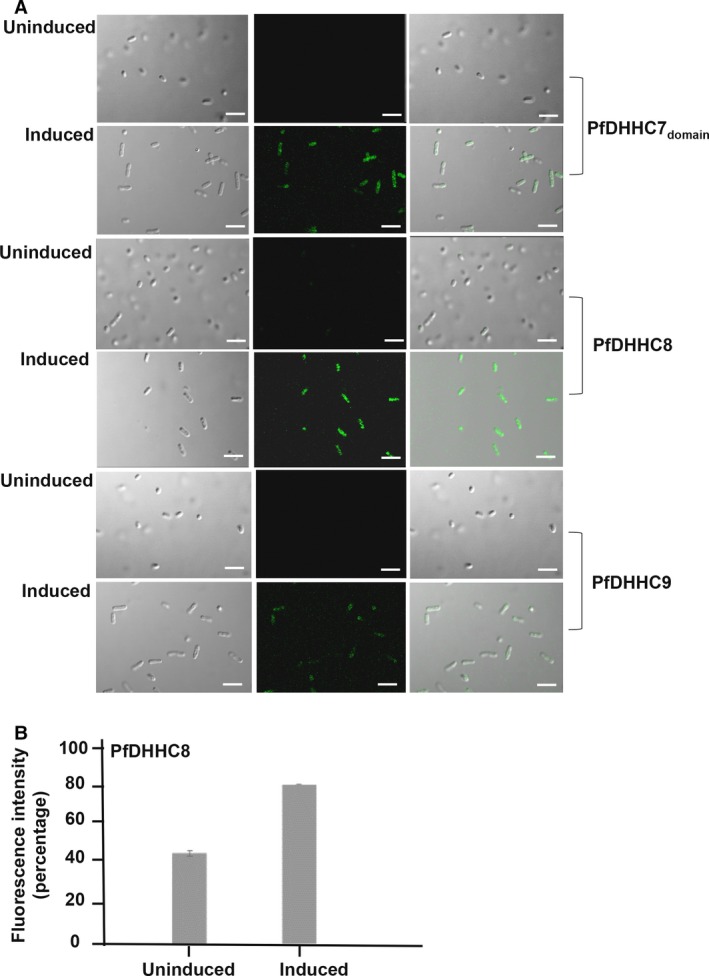
(A) Click chemistry in recombinant PfDHHC‐*E. coli* shows that there is an increase in palmitoylation upon induction, followed by a subsequent reduction upon inhibition. Scale bar indicates 1 μm. (B) Fluorimetry results of click chemistry in PfDHHC‐*E. coli* systems (PfDHHC8). The data represent SD.

As *E. coli* is a PTM‐null system, we tried to visualize the dynamic palmitoylation process using click chemistry with PfDHHCs acting as molecular baits. These proteins enforced transfer of labeled palmitic acid analogues (C‐16 ODYA) to the susceptible cysteine clusters within the *E. coli* proteome, thus quantifying the level of palmitoylation (Refer to Methods). Our results demonstrated increased incorporation of palmitic acid analogues into PfDHHC7 and 8 expressing *E. coli* following induction, as evident by increased fluorescence intensity (Fig. 4A). In case of PfDHHC9‐*E. coli* system, upon induction the fluorescence intensity was found to be minimum thus, suggesting differential activity of PfDHHCs (Fig. 4A). To further validate the acyl‐transferase activity of PfDHHC proteins, we used a known inhibitor of global palmitoylation, 2‐BMP. Upon inhibition, palmitoylation was found to be affected in PfDHHC8 as seen by reduced fluorescence intensity of Oregon green in fluorimetry analysis (Fig. 4B). Based on the microscopy and fluorimetry results, it is confirmed that PfDHHC8 can efficiently induce palmitoylation within the *E. coli* proteome.

### Dynamic palmitoylation showed no detrimental effects on growth of *E. coli* expressing PfDHHCs

**Figure 5:**
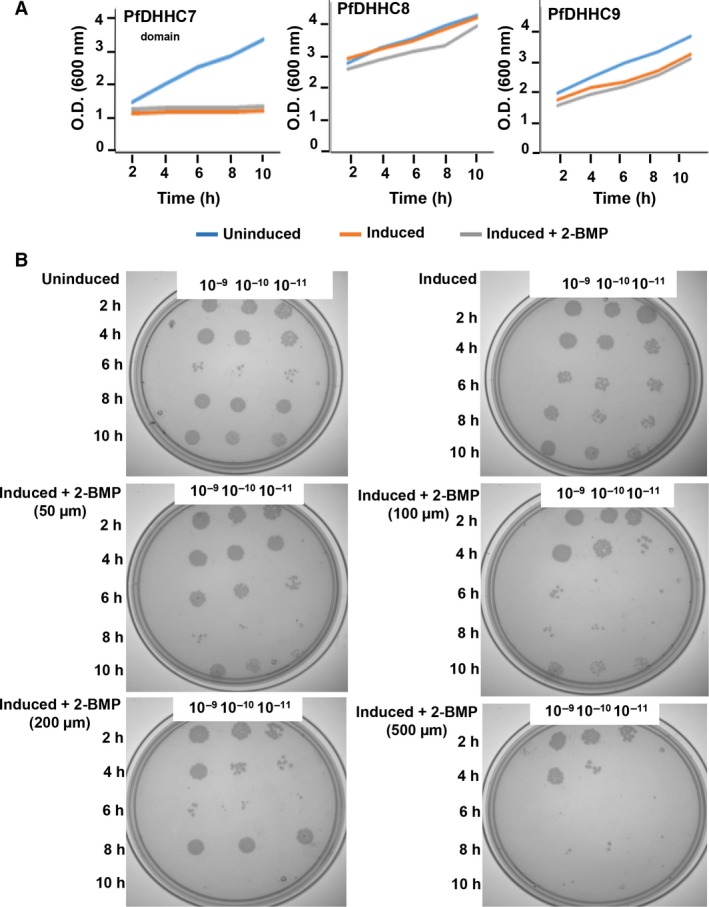
(A) Impact of 2‐BMP inhibition (50 μm) on growth pattern of PfDHHC‐*E. coli* systems demonstrated no significant difference in none of the three PfDHHC‐*E. coli* systems. (B) CFU assay of *E. coli*‐PfDHHC8 strains revealed drastic reduction in CFUs at a concentration ≥ 100 μm.

We evaluated the effects of dynamic palmitoylation and impact of 2‐BMP inhibition on the *E. coli* survival. The results demonstrated no considerable change in growth pattern of cells in both PfDHHC8/9‐*E. coli* systems (Fig. 5A). In case of PfDHHC7‐*E. coli* system, a mild change in growth pattern of *E. coli* was observed (Fig. 5A). Further treatment with 2‐BMP at varying concentrations in induced *E. coli*‐PfDHHC8 cells resulted in no drastic change in growth pattern up to 100 μm, beyond which CFUs were found to be drastically reduced at different titers and in a time‐dependent manner (Fig. 5B).

### Analysis of dynamic palmitoylation in 2‐BMP treated induced PfDHHC8‐*E. coli* system demonstrated reduction in palmitic acid incorporation

**Figure 6:**
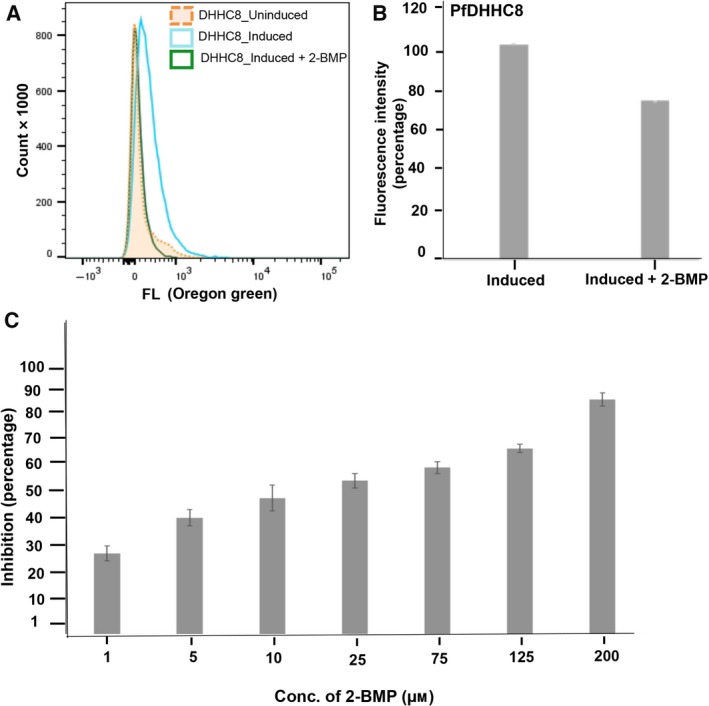
(A) Flow cytometric analysis of 2‐BMP‐based inhibition on global palmitoylation of *E. coli* expressing PfDHHC8. Reduction in uptake of palmitic acid analogue labeled with Oregon green was detected in *E. coli* expressing PfDHHC8. The histograms represent absolute cell counts correlating Oregon green incorporation. (B) Fluorimetry analysis of 2‐BMP‐based inhibition of palmitoylation in *E. coli*‐PfDHHC8 system. (C) SYBR green‐based growth inhibition assay of *Plasmodium* upon 2‐BMP inhibition. Percent inhibition represented dose‐dependent inhibition of palmitoylation with 2‐BMP. The data represent SD.

We tested the alteration in dynamic palmitoylation upon treatment with 2‐BMP postinduction in order to validate the effect of inhibitor in *E. coli* expressing PfDHHC8. The flow cytometric analysis revealed substantial changes in Oregon green intensity. We have also evaluated the changes in palmitoylation using fluorimetry analysis upon treatment with 2‐BMP following induction in the PfDHHC8‐*E. coli* system. The findings revealed ~30% reduction in palmitoylation on treatment in case of the PfDHHC8‐*E. coli* system (Fig. 6A,B).

### 2‐BMP‐based inhibition of global palmitoylation imposed drastic changes in parasite growth

To further evaluate the effects of 2‐BMP on growth progression of *P. falciparum* 3D7, we performed growth inhibition assay. The results demonstrated 50% inhibition at 25 μm concentration of 2‐BMP according to the formula (as described in Methods), as evident by change in SYBR green intensity (Fig. 6C).

### *In silico* prediction of a possible palmitoylome in *E. coli*

**Figure 7:**
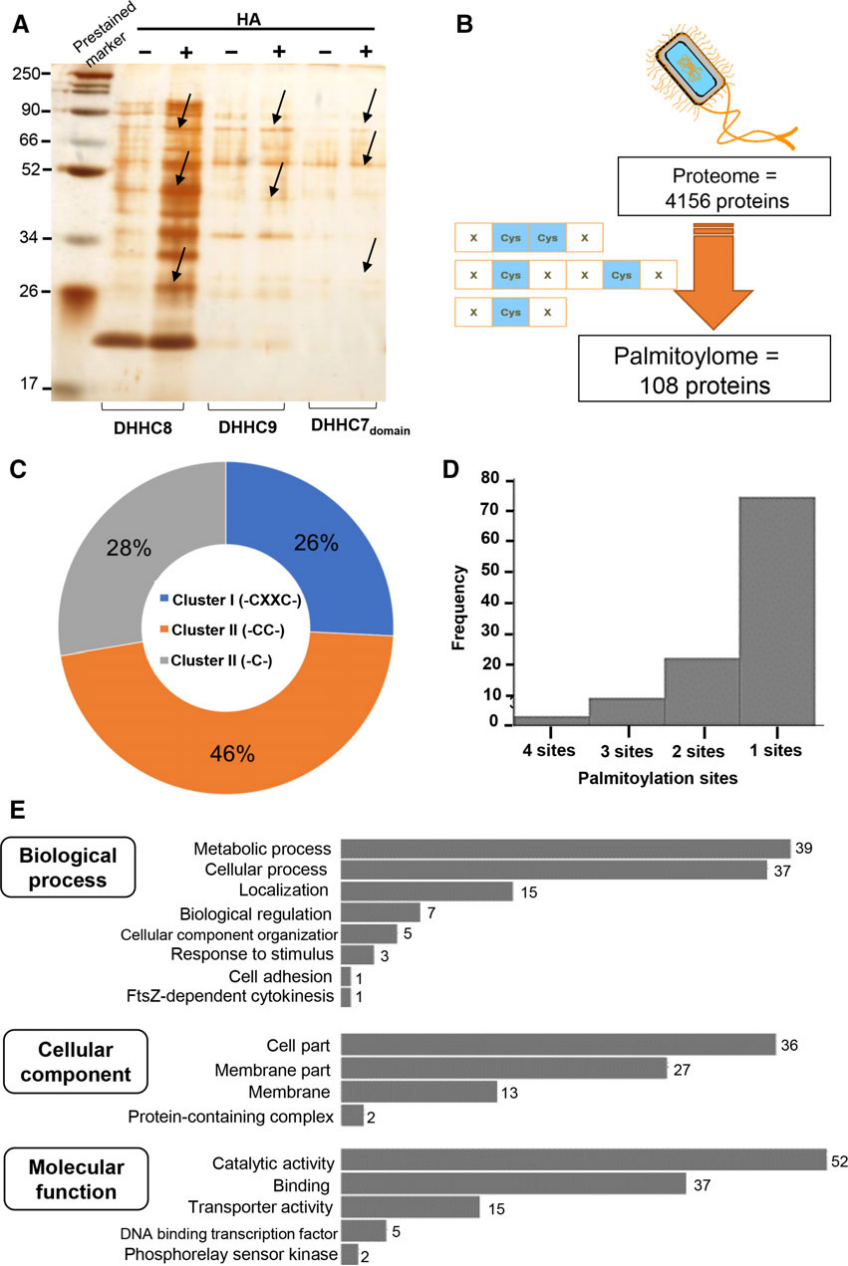
(A) ABE purification of whole proteome of individual PfDHHCs‐*E. coli*. (B) Scheme showing the *in silico* strategy for elucidation of potential palmitoylome in *E. coli*. (C) Pie chart showing the proportion of different palmitoylation site types in 108 proteins. (D) Histogram plot showing the frequency of palmitoylation per protein within the candidate palmitoylome (108). (E) GO analysis plots showing enriched various important GO biological process, cellular component, and molecular function terms among 108 proteins.

To further establish the putative palmitoylome of *E. coli*, we adopted a combinatorial approach comprising ABE and *in silico* analyses. To achieve this, we have induced engineered *E. coli* expressing PfDHHCs along with hydroxylamine (HA) treatment. This led to increased protein palmitoylation in *E. coli* as evident in +HA samples as compared to the ‐HA (Fig. 7A). Among the PfDHHC‐*E. coli* systems, PfDHHC8 showed distinct changes in the palmitoylation profile. On the basis of these findings, we tried to characterize the putative palmitoylome in *E. coli* using *in silico* methods. The results ascertained the possible palmitoylome of *E. coli* which comprised of only 108 proteins containing at least one site prone to palmitoylation using a GPS‐based algorithm (Fig. 7B). Upon further dissection, this set of proteins were found to contain palmitoylation clusters of three categories (I, II, and III) in varying proportions. The highest percentage of sites belonged to cluster II (46%), followed by 26% and 28% within the category of clusters I and III (Fig. 7C). Furthermore, the palmitoylome (*n* = 108) was found to contain maximum of four sites per protein, and the highest number of proteins (*n* = 96) contains only 1–2 probable palmitoylated sites (Fig. 7D).

To understand the role of palmitoylated protein clusters, we performed Gene Ontology analysis and found that the enriched GO Biological processes include metabolic pathways, membrane localization, biological regulation, etc. (Fig. 7E). The second category of GO Cellular component represented enriched majorly GO terms like cell and membrane parts. In case of the GO Molecular function module, GO terms like catalytic activity and transporter activity were enriched (Fig. 7E). Further analysis of their subcellular localization of 108 proteins demonstrated 12 proteins to be located specifically in the cytoplasm, five in F‐complex, four in inner membrane, nucleoid, etc. (Fig. S1C).

### Downstream analyses predicted the involvement of critical protein clusters in *E. coli*

**Figure 8:**
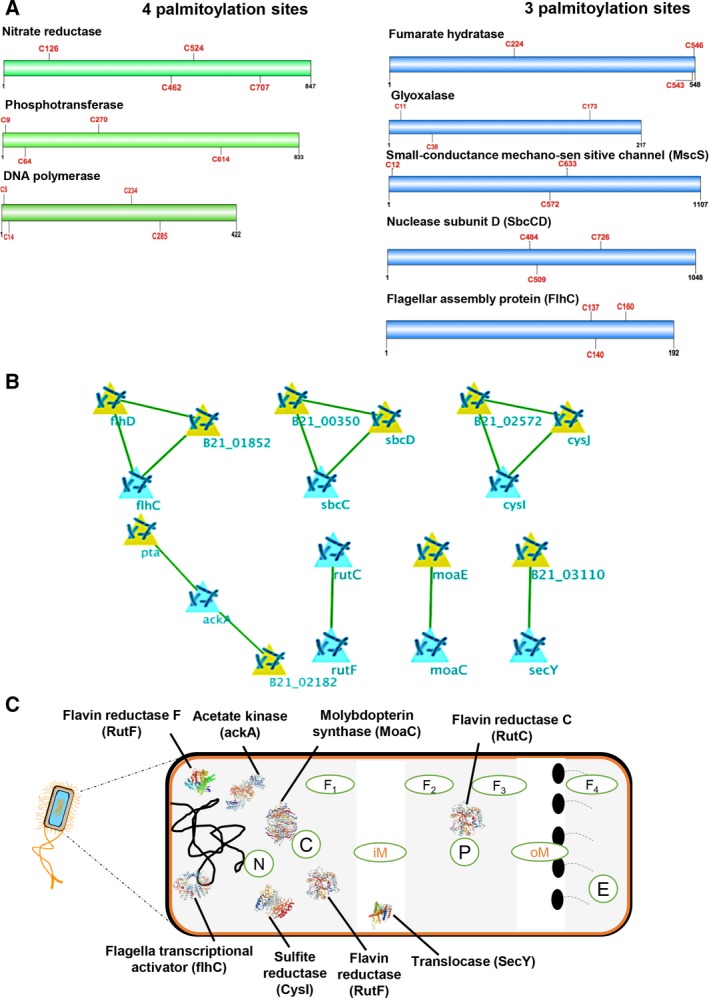
(A) Analysis of specific cysteine sites prone to palmitoylation in proteins containing highest number sites like four sites and three sites. (B) Protein–protein interaction network of highest confidence (> 0.9) showing first‐level interaction with crucial *E. coli* proteins. (C) Cellular localization distribution of eight enriched proteins filtered out of the network analysis.

We further analyzed the frequently palmitoylated protein clusters. Proteins containing three cysteine sites included flagella transcriptional regulator (flhC), rRNA methyltransferase (ksgA), sulfite reductase (cysI), mechanosensitive ion channel (moaC), and nucleases (sbcCD; Fig. 8A). In case of the proteins containing four sites, other essential bacterial proteins like DNA‐dependent DNA polymerase, phosphotransferase and nitrite reductase were enriched (Fig. 8A). These findings suggest that *E. coli* contains essential proteins with specific cysteines which might be prone to palmitoylation.

Upon analyzing the putative protein interaction network (PPI) of the candidate list (*n* = 108), eight highly palmitoylated proteins like translocase subunit (secY), flhC, sulfite reductase I (cysI), sbcC, acetate kinase (ackA), and moaC were found to be strongly interacting with primary neighborhood proteins like BL21_03106, flhD, cysD, and sbcD, respectively (Fig. 8B). Among this set of eight proteins, flhD, SecY, and CysI were found to be specific to nucleoid, inner membrane and outer membrane complex of the *E. coli* cell respectively, whereas proteins sbcC and ackA were found to be localized in the cytoplasm (Fig. 8C). This renders more clarity on the type of proteins which could be affected by induction of palmitoylation in the synthetic *E. coli* cells.

## Discussion

Synthetic biology has provided a wider platform to exploit inherent biological processes for developing high‐throughput drug screening platforms 31. Lipid‐based PTMs are known to play an important role in eukaryotic parasites; however, they have not been exploited so far for designing novel chemotherapeutics 4, 32. In this study, we have engineered *E. coli* for functional validation of three *Plasmodium*‐specific DHHC proteins (PfDHHCs).

Since these enzymes are responsible for transfer of acyl group to specific cysteine thiols in the proteins, we tried to enforce palmitoylation of the engineered *E. coli* proteome by using their inherent palmitic acid metabolism. This system not only envisages a tool for functional validation of DHHCs, but also provides a proof of concept to further develop a high‐throughput synthetic platform for screening small molecule libraries.

Toward this, we extracted *P. falciparum* specific 12 DHHC domain‐containing proteins from PlasmoDB. Assessment of phylogenetic relationship among all P.fDHHCs with other apicomplexan DHHC proteins suggested that the D‐H‐H‐C signature motif was highly conserved, while PfDHHCs demonstrated moderate interspecies conservation. These proteins were structurally modeled and their catalytic pockets were analyzed for molecular docking studies with 2‐BMP and PA. Our results suggest that both compounds can bind to PfDHHC proteins as evident by their binding energies and shared interacting residues within the common catalytic pocket. Among the PfDHHCs, 2‐BMP and PA showed the highest binding affinity to PfDHHC8 with binding energies of −6.5 and −6.3 kCal·mol^−1^, respectively. However, 2‐BMP bound PfDHHC6 model demonstrated nonspecific distal binding from the defined DHHC pocket, while PA was found to be bound to the proximal region of the pocket. This discrepancy in binding energies can be attributed to generic nature of 2‐BMP. Interestingly, docking of its active substrate, palmitoyl‐CoA demonstrated its binding in close proximity to the D‐H‐H‐C motif 33.

Among the PfDHHCs, DHHC9 has been previously shown to play a crucial role in *Plasmodium* gametocytogenesis 8. Two other PfDHHCs (7 and 8) are known to be present in the asexual blood stage of the parasite. Specifically, DHHC7 has been reported to be crucial for invasion of its sister parasite, *T. gondii* into the host 13, 34. Further we checked the expression of the cloned PfDHHCs (7, 8, and 9) and also functionally validated them in *E. coli* using click chemistry. The findings suggest that upon induction, engineered *E. coli* strains were likely to utilize their own palmitic acid and enforce palmitoylation of their proteome. To visualize palmitoylation in *E. coli*, we performed click chemistry‐based metabolic labeling. The data suggest that PfDHHC7, PfDHHC8, and PfDHHC9 expressing *E. coli* systems can dynamically incorporate palmitic acid analogues. Since protein palmitoylation is a foreign molecular mechanism in *E. coli*, we thought to use a known inhibitor, 2‐BMP for studying the phenotypic effects. Notably, 2‐BMP could reverse the palmitoylation status of *E. coli* without significant morphological changes. Furthermore, induced PfDHHC8‐*E. coli* showed no changes in growth pattern upon 2‐BMP inhibition up to 100 μm, whereas at higher concentration up to 500 μm, there was drastic reduction in CFUs observed in a time‐dependent manner.

**Figure 9:**
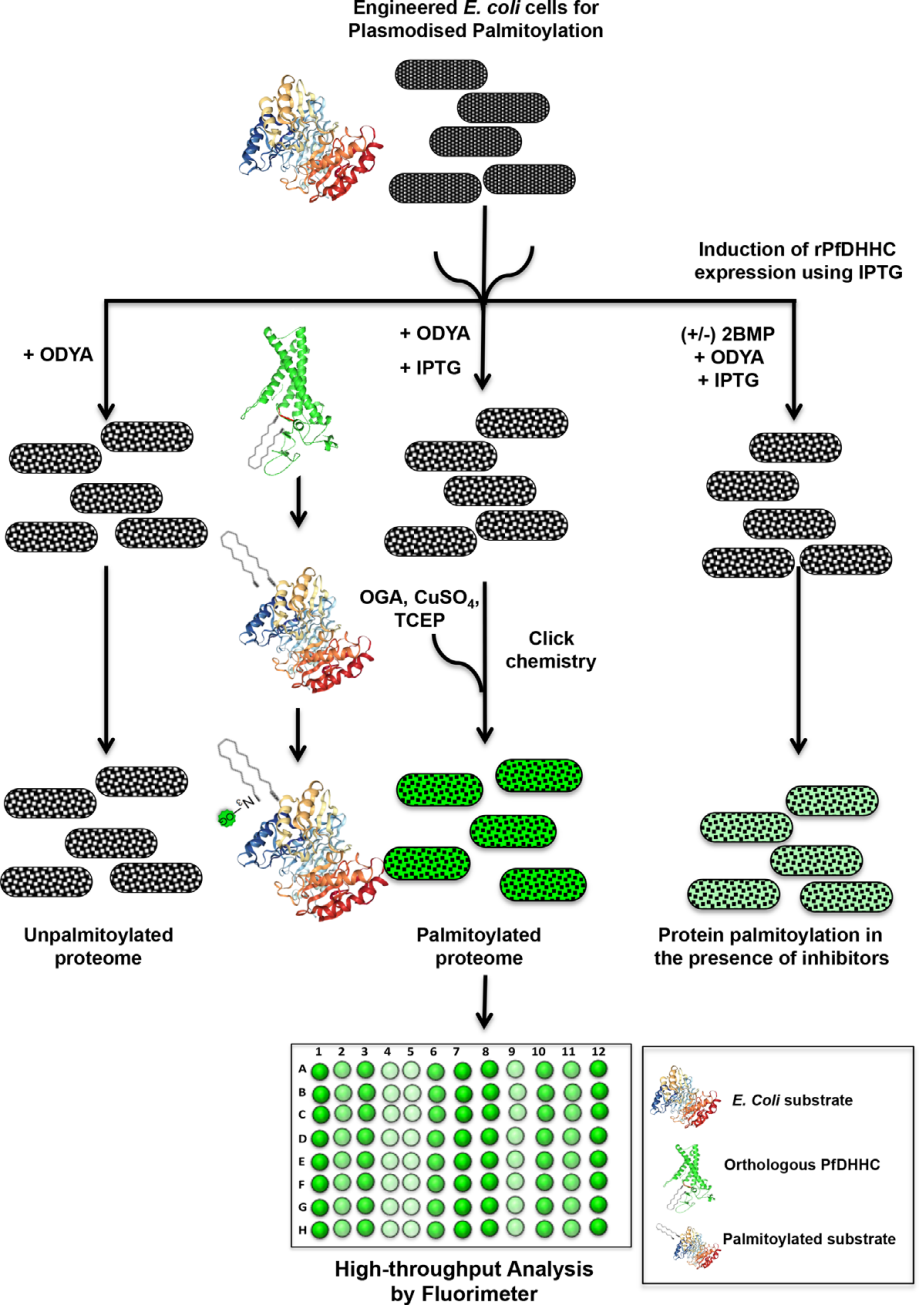
Schematic model of *Plasmodium falciparum* palmitoylation machinery engineered *E.coli* drug screening system

Since the *E. coli* proteome comprises of 4156 proteins, we decided to evaluate its predictive palmitoylome using *in silico* tools. Based on a Group prediction system‐based algorithm, we could predict palmitoylation sites and generate a catalog of 108 palmitoylated proteins. Analysis of these palmitoylation sites indicated the category of cysteine‐rich clusters. Albeit the highest number of palmitoylated sites were found to be four in three individual proteins, the majority of *E. coli* proteins (96 out of 108) only contained 1–2 palmitoylation sites. Therefore, there is a possibility that the impact of palmitoylation might not substantially affect growth of the bacteria. Furthermore, we propose that the development of a drug screening system based on this machinery would be an efficient tool to target the DHHC proteins of *Plasmodium* and can be subsequently extended to other pathogens (Fig. 9). In summary, our proposed model of engineered *E. coli* mimicking *Plasmodium*‐specific palmitoylation can be utilized as a high‐throughput drug screening platform against tropical parasites.

## Author contributions

SS conceptualized, designed all the experiments, and analyzed the data. RA and SP planned the computational strategy. RA performed all computational analyses, while RJ did the docking studies. RS conducted the growth inhibition experiments in *P. falciparum*. PY characterized the recombinant protein, performed click chemistry and ABE assay in *E. coli*. SG, NJ, and PY performed FACS and microscopy. SP, RA, SG, and SS wrote the manuscript with analytical input from SS.

## Conflict of interest

The authors declare no conflict of interest.

## Supporting information


**Fig. S1.** Evolutionary conservation analysis of PfDHHCs among apicomplexan family, structural analysis of ligandability of PfDHHCs toward palmitoyl‐CoA, and predicted palmitoylome of *E.coli*, the PTM‐null system. (A) Interspecies phylogenetic analysis showing moderate conservation across apicomplexan parasite protein families namely, *Plasmodium falciparum, Plasmodium berghei, Toxoplasma gondii, Babesia bovis*,* Theileria parva*,* Cryptosporidium muris* and *Eimeria tenella*. (B) Molecular docking of substrate intermediate palmitoyl‐CoA and PfDHHC8 showed binding affinity to overall pocket but not specific to the DHHC motif of protein (Energy = −6.3 kCal·mol^−1^). (C) Cellular localization distribution of all 108 palmitoylated proteins.
