# Supplementary figures and images for: Plasmodium palmitoylation machinery engineered in E. coli for high‐throughput screening of palmitoyl acyl‐transferase inhibitors
Source: FEBS Open Bio. 2019 Jan 10;9(2):248–64. doi: 10.1002/2211-5463.12564 (PMC6356172; doi:10.1002/2211-5463.12564)

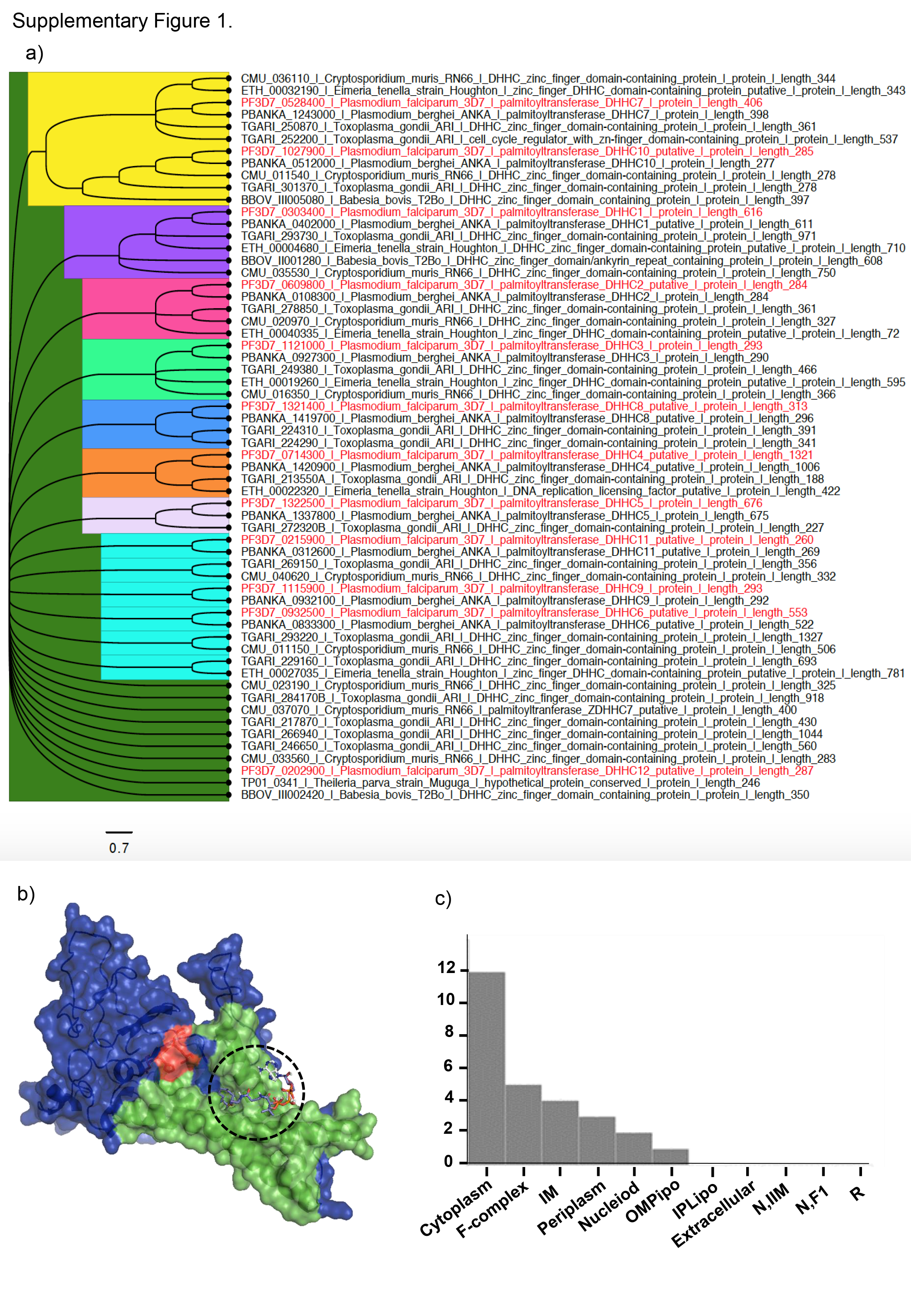

Supplement: Supplementary file 1 — Fig. S1. Evolutionary conservation analysis of PfDHHCs among apicomplexan family, structural analysis of ligandability of PfDHHCs toward palmitoyl‐CoA, and predicted palmitoylome of E.coli, the PTM‐null system. (A) Interspecies phylogenetic analysis showing moderate conservation across apicomplexan parasite protein families namely, Plasmodium falciparum, Plasmodium berghei, Toxoplasma gondii, Babesia bovis, Theileria parva, Cryptosporidium muris and Eimeria tenella. (B) Molecular docking of substrate intermediate palmitoyl‐CoA and PfDHHC8 showed binding affinity to overall pocket but not specific to the DHHC motif of protein (Energy = −6.3 kCal·mol−1). (C) Cellular localization distribution of all 108 palmitoylated proteins. [file FEB4-9-248-s001.tif]
